# Three-dimensional culture in a bioengineered matrix and somatic cell complementation to improve growth and survival of bovine preantral follicles

**DOI:** 10.1007/s10815-025-03497-3

**Published:** 2025-05-20

**Authors:** Juliana I. Candelaria, Ramon C. Botigelli, Carly Guiltinan, Ariella Shikanov, Anna C. Denicol

**Affiliations:** 1 Department of Animal Science, University of California Davis, Davis, CA USA; 2 Department of Biomedical Engineering, University of Michigan, Ann Arbor, MI USA; 3 Department of Obstetrics and Gynecology, University of Michigan, Ann Arbor, MI USA; 4 Department of Macromolecular Science and Engineering, University of Michigan, Ann Arbor, MI USA; 5 Current address: Department of Biochemistry and Molecular Biology, Uniformed Services University of the Health Sciences, Bethesda, MD USA; 6 Department of Biomolecular Engineering, University of California Santa Cruz, Santa Cruz, CA USA

**Keywords:** Preantral follicle, Three-dimensional culture, Embryonic stem cells, Ovary, Bovine, Biomimetic

## Abstract

**Purpose:**

Here, we explored poly(ethylene glycol) (PEG) bioengineered hydrogels for bovine preantral follicle culture with or without ovarian cell co-culture and examined the potential for differentiation of bovine embryonic stem cells (bESCs) towards gonadal somatic cells to develop a system better mimicking the ovarian microenvironment.

**Methods:**

Bovine preantral follicles were first cultured in two-dimensional (2D) control or within PEG hydrogels (3D) and then co-cultured within PEG hydrogels with bovine ovarian cells (BOCs) to determine growth and viability. Finally, we tested conditions to drive differentiation of bESCs towards the intermediate mesoderm and bipotential gonad fate.

**Results:**

Primary follicles grew over the 10-day culture period in PEG hydrogels compared to 2D control. Early secondary follicles maintained a similar diameter within the PEG while control follicles decreased in size. Follicles lost viability after co-encapsulation with BOCs; BOCs lost stromal cell signature over the culture period within hydrogels. Induction of bESCs towards gonadal somatic fate under WNT signaling was sufficient to upregulate intermediate mesoderm (*LHX1*) and early coelomic epithelium/bipotential gonad markers (*OSR1*, *GATA4*, *WT1*). Higher BMP4 concentrations upregulated the lateral plate mesoderm marker *FOXF1*. *PAX3* expression was not induced, indicating absence of the paraxial mesoderm lineage.

**Conclusions:**

Culture of primary stage preantral follicles in PEG hydrogels promoted growth compared to controls; BOCs did not maintain identity in the PEG hydrogels. Collectively, we demonstrate that PEG hydrogels can be a potential culture system for early preantral follicles pending refinements, which could include addition of ESC-derived ovarian somatic cells using the protocol described here.

**Supplementary Information:**

The online version contains supplementary material available at 10.1007/s10815-025-03497-3.

## Introduction

Folliculogenesis is a selective process resulting in production of a limited quantity of competent oocytes over a female’s lifespan. The processes that coordinate the activation and growth of ovarian follicles in non-rodent species have yet to be fully elucidated, especially during the early preantral stages of development; however, progress has been hindered by the lack of appropriate in vitro culture systems and an insufficient knowledge base of ovarian development. A deeper understanding of preantral folliculogenesis is critical to enable in vitro folliculogenesis beginning at the preantral stage and to establish alternative fertility preservation methods by utilizing the preantral follicle pool for oocyte production [1]. This approach would be beneficial for prepubertal girls suffering from blood-borne malignancies as isolated preantral follicles could be matured in vitro without the risk of reintroducing malignant cells or surgical intervention, as could be the case with reintroduction of ovarian tissue fragments.

Recapitulation of bovine preantral folliculogenesis as a model for humans has been attempted by several groups, but there has been little success in efficiently growing follicles to the preovulatory stage and yielding metaphase II oocytes. Although 2-dimensional culture systems have shown some promise in supporting bovine preantral follicle growth [2–4], mounting evidence suggests that 3-dimensional (3D) culture systems better promote development due to their ability to maintain a follicle’s spherical configuration and preserve cell contact both within the follicular unit (i.e., granulosa cell and oocyte interface) and outside the basement membrane where theca cells are located [5–8]. Matrigel and alginate are commonly used biomaterials to create hydrogels for in vitro follicle culture; however, pitfalls include uncontrollable degradation and biological inertness, respectively [9]. Therefore, a highlighted alternative is the use of bioengineered 3D hydrogel culture systems that permit timely degradation by the follicle while retaining the mechanical support necessary to keep its spherical structure [10, 11]. Poly(ethylene glycol) (PEG) hydrogels constructed with proteolytically degradable peptide crosslinkers have been employed for mouse in vitro folliculogenesis and demonstrated success in growing preantral follicles to the antral stage and producing metaphase II oocytes [12]. Likewise, the addition of adipose-derived stem cells or conditioned media to PEG hydrogels containing mouse preantral follicles further aided development and survival [13]. Despite the promise demonstrated in mice, the use of PEG hydrogels for large mammal in vitro preantral follicle culture has not been reported. Moreover, investigating cell supplementation in the hydrogel system to determine their propensity to become theca cells and/or advantageous effect on follicle growth has not been directly assessed. Indeed, early preantral follicles do not contain theca cells and must recruit precursor cells from the stromal environment to build the essential theca layer; hence, the addition of cells to the system would be essential when starting with primordial- or primary-stage follicles.

Although native ovarian stromal cells have been used as feeder cells for 2D and 3D in vitro follicle culture [14–16], surgical intervention is required to obtain ovarian tissue and harvest cells. Moreover, their abilities to phenotypically-mirror theca cells upon co-culture with follicles have not been shown to date. Alternative cell sources, particularly those of renewable capacity (i.e., stem cells), present a unique advantage to generating cells that could give rise to a theca cell-like phenotype. To that end, stepwise in vitro differentiation of pluripotent stem cells into gonadal-like cells has been demonstrated in humans and mice [17–19] thus highlighting the potential to create a substitute cell source with prospective theca cell characteristics. Cell fate decisions that form the bipotential gonad are the culmination of precisely timed cell signaling via morphogens such as FGF, BMP, and WNT proteins; hence, stepwise induction protocols that replicate these signaling events are used in in vitro systems [20–22]. To date, methods to guide in vitro cell differentiation of bovine embryonic stem cells have not been reported.

In this study, we describe the use of a PEG hydrogel culture system with or without native ovarian cells for in vitro development of bovine preantral follicles and explore the potential of bovine embryonic stem cells to be induced towards gonadal-like cells in vitro. We first hypothesized that culture in PEG hydrogels would improve the growth and viability of bovine preantral follicles. By examining the gene expression of ECM-degrading enzymes of primary- and early secondary-stage follicles, we tailored the hydrogel design to respond to bovine follicle enzyme secretion. Based on previous evidence that cell supplementation improves mouse follicle development, we also examined the effect of dissociated bovine ovarian cells (BOCs) on bovine preantral follicle growth and if BOCs exhibit a pre-theca cell phenotype when cultured in vitro using PEG hydrogels. Finally, as a first-step approach to ultimately create theca-like cells for preantral follicle co-culture, we tested the differentiation of bovine embryonic stem cells (bESCs) into the intermediate mesoderm and early coelomic epithelium/bipotential gonad-like cells in vitro.

## Material and methods

All materials were purchased from Thermo Fisher Scientific unless otherwise specified.

### Follicle isolation and bovine ovary single-cell dissociation

Fresh bovine preantral follicles were isolated as previously described [23]. Follicles were maintained in warmed follicle wash media until subsequent experiments. For bovine ovary single-cell dissociation (*n* = 3 replicates), cortical tissue (250–300 mg) was fragmented into 500 µm^2^ pieces incubated with Hank’s balanced salt solution (Ca^2+^/Mg^2+^) containing 1 mg/mL collagenase IV, 1 U/mL DNase I, and 50 µg/mL Liberase (5.401.119.001, Sigma-Aldrich). The solution with tissue was shaken at 38.5 °C for 20 min, pipetted 15 times, and shaken again for 20 min to mechanically aid in the dissociation of cells. Enzymatic activity was stopped by adding 20% v/v fetal bovine serum to the solution. The tissue-dissociated solution was filtered through a 100-µm and a 35-µm cell strainer. Cells collected after final filtration were centrifuged (300 × *g*, 5 min) and the resulting pellet was resuspended in 1 mL Hank’s balanced salt solution without Ca^2+^/Mg^+^ until encapsulation.

### PEG hydrogel materials, preparation, and follicle encapsulation

PEG hydrogel experiments were conducted using 8-arm PEG vinyl sulfone (PEG-VS) (40 kDa, > 99% purity, JenKem Technology) and crosslinked with MMP- and Plasmin-sensitive peptide sequences (Ac-GCRD**VPMS**MRGGDRCG**YKNS**CG, i.e., YKNS/VPMS) (2391.8 g/mol, > 90%, Celtek Peptides). PEG and crosslinker peptide were dissolved in isotonic 50 mM HEPES buffer and mixed to a final composition containing 5% PEG-VS with 1:1 stoichiometric ratio of VS to thiol (side chain present on cysteines in peptide sequence). Sixteen-µL hydrogels containing preantral follicles were formed between parafilm-lined glass slides and allowed to gelatinize for up to 10 min. Then, hydrogels were transferred to warmed follicle maintenance media to swell and placed in a humidified incubator (37 °C). Freshly isolated follicles were maintained in warmed follicle wash medium until encapsulation. In experiment 1, follicles (*n* = 1–4) were first washed in 10% PEG-VS and either encapsulated in PEG hydrogels or placed into 2D culture. Hydrogel-encapsulated and 2D culture follicles were placed into individual wells of a 96-well plate containing follicle culture medium (1:1 ratio of α-MEM and F12 with glutamine supplemented with 3 mg/mL bovine serum albumin (BSA), 100 ng/mL penicillin/streptomycin, 1 mM sodium pyruvate, 1% (v/v) non-essential amino acids, 10 mg/L insulin, 5.5 mg/L transferrin and 6.7 µg/L selenium (ITS), 100 ng/mL human recombinant follicle stimulating hormone (228–12,609-2, RayBiotech), and 50 µg/mL ascorbic acid). Half of the spent media was changed every 2 days and follicles were cultured for 10 days. To assess follicle growth, the average of 2 perpendicular diameter measurements was taken at day 0, day 5, and day 10 using an inverted microscope (Revolve, Echo Labs). The percentage of growing and non-growing follicles (growth defined as having at least 5% increase in diameter by day 10 compared to day 0) and percent increase in size in growing-only follicles was calculated. In total, 15 replicates were conducted with a total of 236 follicles (*n* = 153 in PEG and *n* = 83 in 2D control).

### Cell viability staining and quantification

Bovine ovarian cells were resuspended in PEG-VS + crosslinker peptide VPMS/YKNS at 1, 2, 10, and 20 × 10^6^ cells/mL to test cell viability immediately after encapsulation, 5 and 10 days after culture. Hydrogels were cultured individually in 96-well plates with 150 µL of follicle culture media with half media changes every 2 days. After culture, hydrogels were incubated for 10 min with pre-equilibrated alpha-MEM containing 2 µM propidium iodide (PI) and 1:1000 Hoechst (1 µg/mL final concentration). Negative control hydrogels were void of propidium iodide and used for setting exposure during imaging. Hydrogels were imaged using an epifluorescent microscope (Revolve) and the total number of cells and viable cells were quantified using ImageJ after converting to binary images where PI/Hoechst-positive cells were white and background was black. A total number of cells positive for PI were divided by total cells quantified by Hoechst staining to determine percentage of unviable cells. The experiment was repeated 4 times using 3 hydrogels per cell density for each replicate.

### Encapsulation and co-culture of bovine ovarian cells and preantral follicles in PEG

To test the impact of BOC and co-encapsulation on follicle viability and growth and theca cell differentiation during in vitro culture, hydrogels were formed with either (1) only follicles, (2) 5 × 10^6^ BOCs/mL only, or (3) follicles and 5 × 10^6^ BOCs/mL BOCs. Each hydrogel contained 1–5 follicles (*n* = 65 follicles; 5 replicates). Hydrogel formation and follicle encapsulation were performed as previously described with the addition of staining follicles with the cell membrane marker PKH26 (2 µM final concentration; Sigma-Aldrich) prior to encapsulation to improve visualization. Hydrogels were cultured for 10 days with half of the medium changed every other day. Follicles were considered viable if the basement membrane was attached, the follicles were void of vacuoles, and granulosa cells were not darkened or mishappened. To recover cells for RT-qPCR, the hydrogels were incubated in phenol red-free alpha-MEM with Liberase (1.3 wünsch units/mL) and incubated at 37 °C and 5% CO_2_ for 30 min with repeated pipetting to degrade the hydrogel. After centrifugation, the cell pellet was used for RNA extraction, cDNA synthesis, and RT-qPCR as described below.

### Culture of bovine embryonic stem cells (bESCs)

Bovine embryonic stem cells (*n* = 2 female lines) [24] were routinely cultured feeder-free in NBFR medium (N2B27 medium (1:1 DMEM/F12 and Neurobasal media, 0.5% v/v N-2 supplement, 1% v/v B-27 supplement, 2 mM MEM non-essential amino acid solution, 1% v/v GlutaMAX supplement, 0.1 mM 2-mercaptoethanol, 100 U/mL penicillin, and 100 µg/mL streptomycin) supplemented with 1% BSA, 20 ng/mL bFGF (100-18B, PeproTech), 2.5 µM IWR-1 (I0161, Sigma), and 20 ng/mL activin A (338-AC, R&D Systems). Cells were passaged onto vitronectin-coated plates every 2–4 days using TrypleExpress at 1:4–1:6 split ratio and cultured in humidified incubators at 37 °C and 5% CO_2_. When cells were passaged, media was supplemented with 10 µm ROCK inhibitor Y-27632. Media was changed daily and bESCs used for mesoderm induction were between passage 18 and 26.

For mesoderm induction, bESCs were passaged onto 12-well vitronectin-coated plates at 40,000 cells per well in mesoderm-induction media (GMEM medium, 15% knockout serum replacement, 0.1 mM NEAA, 2 mM GlutaMAX, 1 mM sodium pyruvate, 0.1 mM β-mercaptoethanol, 100 U/mL penicillin, and 100 µg/mL streptomycin and supplemented with 3 µm CHIR99021 and 70 ng/mL activin A). Cells were cultured in mesoderm induction medium for 48 h with daily medium change, and thereafter, the cells were considered “mesoderm-like cells” (MeLCs) and used for subsequent experiments.

Mesoderm-like cells were cultured in mesoderm induction medium without activin A for an additional 72 h and supplemented with 10 ng/mL bFGF beginning immediately after MeLC induction, 24 h after MeLC induction, 48 h after MeLC induction, or no supplementation (mesoderm medium only). Based on the finding of which bFGF regimen best upregulated gene expression, in a second experiment, MeLCs were cultured in mesoderm induction media without activin A, with 10 ng/mL bFGF and with either 0, 1, 10, or 20 ng/mL of BMP4 for an additional 72 h. All experiments contained duplicate wells for each condition tested and had 3 biological replicates/independent differentiations. Cells were imaged every day during differentiation on the EVOS (Thermo Fisher) inverted microscope.

### End-point (RT-PCR) and quantitative reverse transcription PCR (qRT-PCR)

Freshly isolated follicles were pooled by stage and snap frozen in minimal PBS. To examine the expression of transcripts for extracellular-matrix degrading enzymes, primary follicles (*n* = 3 pools of 46–56 follicles) and early secondary follicles (*n* = 3 pools of 34–40 follicles) were subjected to RNA isolation using a protocol with a combination of Trizol, Purelink DNase treatment, and Qiagen RNeasy Micro Kit. Final total RNA was eluted into 14 µL of RNase-free water. Bovine ovarian cells (BOCs), bESCs, MeLCs, and differentiated cells were pelleted, snap frozen, and subjected to RNA isolation using the Qiagen RNeasy Micro Kit. Maximum available RNA mass was used for cDNA synthesis of primary and early secondary follicles; for cells, 250 ng–1 µg of total RNA was used for cDNA synthesis. For RT-qPCR, 1–2 µL of cDNA (50 ng total), 1 × SsoAdvanced Universal SYBR Green Supermix (Bio-Rad, Hercules, CA), and 250 nM of forward and reverse primers were mixed for 10 µL total volume. The reaction was carried out in the CFX96 Real-Time PCR Detection System (Bio-Rad). Cycling conditions were an initial denaturation step at 95 °C for 30 s followed by 40 cycles of denaturation at 95 °C for 10 s, annealing at 60 °C for 30 s, and extension at 60 °C for 5 s. To confirm specificity of PCR products for each gene, each assay included a melt curve analysis and non-template control. Data was normalized to *ACTB* by the delta-Ct method and relative expression data are represented as 1/ΔCT. For visualization, PCR products from quantitative PCR reactions were ran on a 2% agarose gel.

For RT-qPCR experiments using *BAX* and *BCL2* primers, a standard curve of fresh bovine ovarian cells was used to estimate absolute quantity of mRNA present in unknown samples of BOCs. The ratio of *BAX*/*BCL2* was calculated from mRNA quantity of respective genes and used to determine if cells were in a pro- or anti-apoptotic state. All primers are listed in Table 1.

**Table 1:** Primer list

Gene	Forward sequence (5′−3′)	Reverse sequence (5′−3′)	Product size (base pairs)
*BAX*	CATGAGGTCAACCCTCGGGA	TAG GGA AGC CCA TGCTGAGT	117
*BCL2*	GAGTTCGGAGGGGTCATGTG	GGG CCA TAC AGC TCCACAAA	158
*CSPG4*	CTGGAGGAACAA AGGTCTCTGG	ACTGCGTGATCTGGAAAAGCA	140
*DHH*	GCATGCATTGCAGGTCGTC	ACGTGGGCAGAATGCTAGTC	145
*FOXF1*	GCCTCCTACATCAAGCAGCA	GTTCTGGTGCAGATACGGCT	108
*GATA4*	TGCGCCCCATCAAAACAGAG	GGGCCATACAGCTCCACAAA	77
*GLI1*	CCTTCAAAGCCCAGTACATGC	GTGCGTCTTCAGATTTTCCAG	122
*IHH*	CGGCTTCGACTGGGTGTATT	GGCTGAGTGCTCGGACTTG	70
*LHX1*	TGGACCGCTTCCTCTTGAAC	CTTGGTACCGAAACACCGGA	152
*MMP2*	ATCGTCTTCGACGGC ATCTC	TTCGCCAGATGAATCGGTCC	70
*MMP9*	CTTCACCTTTGAGGGTCGCT	TGGGTGTAGAGTCTCTCGCT	138
*OSR1*	CCTGTGGAAATCCAGCGCC	CAGTCTGTTCTGACTTCCTGCTTA	111
*PAX3*	CCTTCAAAGCCCAGTACATGC	GTGCGTCTTCAGATTTTCCAG	122
*PLAT*	TCGGCAGGCCCTCTATTCTT	ACGTGGCGGTAGCATCTATTT	89
*PLAU*	TGGGTCAGTCACGGCTTAAC	ACGGATCTTCAGCAAGGCAAT	131
*WT1*	CCTTCACCGTCCACTTCTCT	CCGTGCTGTATCCCTGGTTG	183
*H2A.2*	GAGGAGCTGAACAAGCTGTTG	TTGTGGTGGCTCTCAGTCTTC	104
*ACTB*	CTCTTCCAGCCTTCCTTCCT	GGGCAGTGATCTCTTTCTGC	178

### Statistical analysis

All data were first assessed for normality using a Shapiro–Wilk test. PEG growth data was subjected to a two-way ANOVA using RStudio with effect of replicate included in the model. Cell viability and RT-qPCR data were subjected to a one-way ANOVA or Kruskal–Wallis test if data was not normally distributed using Graph Prism. Post-hoc tests (Tukey’s and Dunn’s for ANOVA and Kruskal–Wallis, respectively) were conducted when appropriate. RT-qPCR data were analyzed based on delta-Ct values. Comparisons were considered significantly different when associated with *P* < 0.05.

## Results

**Fig. 1:**
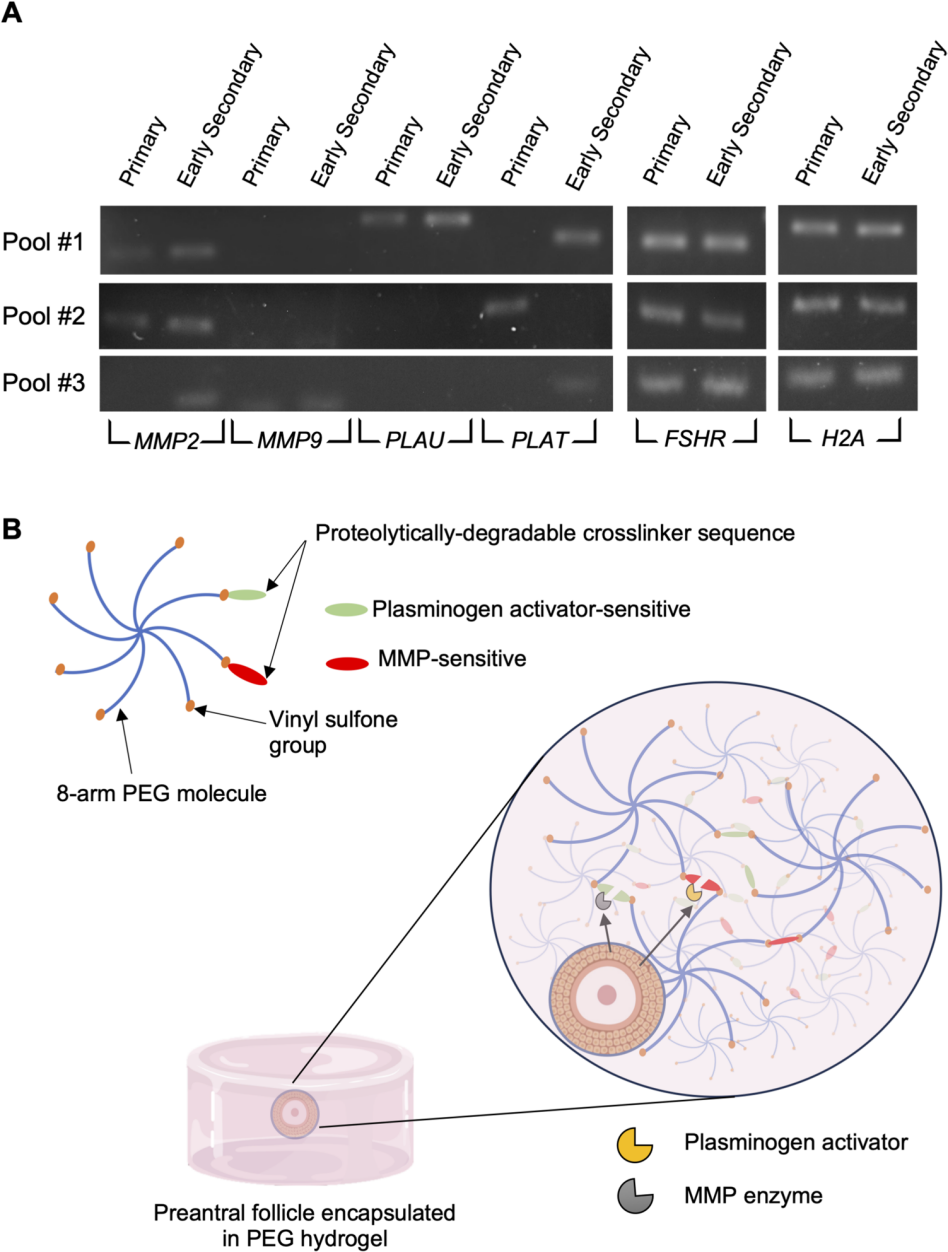
Bovine preantral follicles express extracellular matrix-degrading enzymes, allowing the design of a PEG hydrogel containing target peptides for controlled degradation. **A** Agarose gel with products of RT-PCR of primary and early secondary follicles expressing mRNA for *MMP2*, *MMP9*, *PLAU*, *PLAT*, *FSHR*, and *H2A.* Each pool of primary and early secondary follicles (*n* = 3 pools per stage) contained 46–56 and 34–40 follicles, respectively. **B** Schematic of PEG hydrogel for follicle encapsulation. PEG, poly(ethylene glycol); MMP, matrix metalloproteinase. Figure made with Biorender

To design a proteolytically degradable PEG hydrogel that would most likely be compatible with bovine preantral follicles, we first examined the gene expression of ECM-degrading enzymes in primary and early secondary follicles that express the key follicle marker *FSHR*. Matrix metalloproteinases (MMPs) and plasminogen activators are ECM-degrading enzymes and play a pivotal role in tissue remodeling. We found that bovine primary and early secondary follicles express *MMP2*, although in one pool, we only found *MMP2* in early secondary follicles. However, neither follicle stage expressed *MMP9* (Fig. 1A)*.* Plasminogen-activator urokinase (*PLAU*) was expressed in primary and early secondary follicles from one pool, but not in the second or third pool. Similarly, plasminogen-activator tissue (*PLAT*) was expressed in primary follicles of one pool and in early secondary follicles from two other pools (Fig. 1A). Based on these results, we proceeded to design a PEG hydrogel that is functionalized with VPMS (MMP-sensitive) and YKNS (plasminogen-sensitive) peptide sequences (Fig. 1B).

**Fig. 2:**
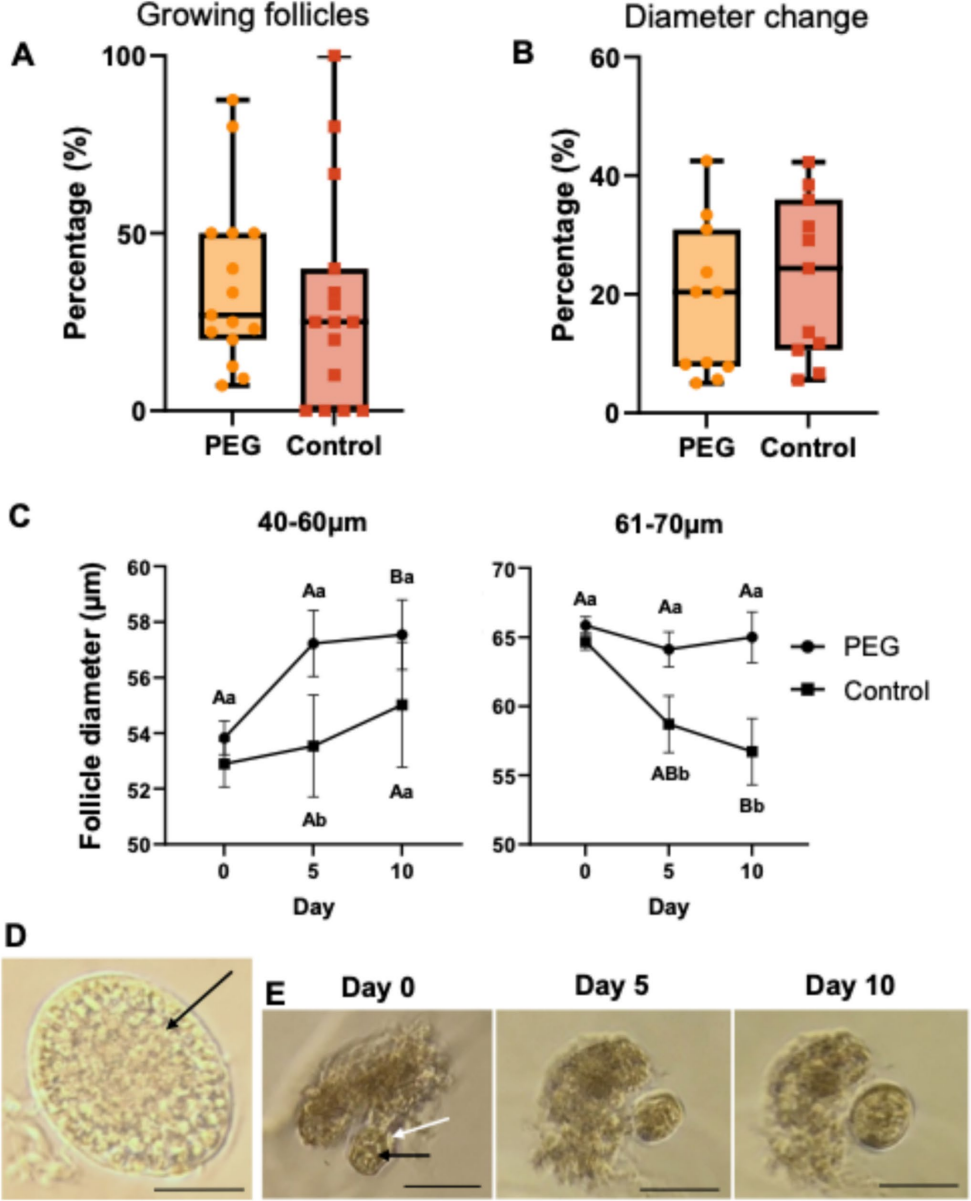
Bovine preantral follicle in vitro growth using PEG or 2D control culture systems. **A** Percentage of preantral follicles that grew (*n* = 236) and **B** percent change in diameter from follicles that grew (*n* = 171). **C** Bovine preantral follicle diameter change (from all follicles cultured; *n* = 236) during in vitro culture at indicated starting diameters. Lowercases letters (a, b) that differ = significant difference (*P* < 0.05) between treatment groups in the same day. Uppercase letters (A, B) that differ = significant difference (*P* < 0.05) within the same treatment group but compared to day 0. Data are presented as mean ± SEM. **D** Representative image of a freshly isolated follicle with a visible oocyte. Scale bar = 50 µm. **E** Representative images of a follicle grown in a PEG hydrogel over the 10-day culture period. White arrow shows the follicle attached to ovarian tissue; black arrows indicate the oocyte. Scale bar = 100 µm

Next, we hypothesized that bovine preantral follicles are more likely to develop when encapsulated in PEG hydrogels compared to 2D traditional methods after 10 days of culture. We found no difference in the percentage of growing follicles between PEG (35.8% ± 6.23%) and 2D control (30.3% ± 7.88%) (*P* = 0.56) (Fig. 2A). Replicate (i.e., individual ovary) affected the percentage of growing follicles (*P* < 0.05) regardless of culture condition. It is worth noting that in the control group, no follicle growth was observed in four of the 15 replicates (approximately 25% of the time), whereas in the PEG group, there was growth, although variable, in every replicate (Fig. 2A). Of the replicates where at least one follicle demonstrated growth in either condition, we found no difference (*P* = 0.50) in the growth rate from initial size (PEG: 18.8% ± 3.32%, control: 22.7% ± 3.49%) (Fig. 2B). There was no effect of replicate on growth rate (*P* = 0.78).

We further examined the diameter changes between day 0, 5, and 10 in follicles within a narrow range of starting diameters corresponding to their stage of development between the primary (40–60 µm) to early secondary (61–70 µm). When we examined follicles with starting diameters corresponding to the primary stage, we found PEG hydrogels outperformed the control conditions as day 5 follicles were larger in PEG compared to 2D control (57.2 ± 1.2 vs 53.5 ± 1.8 µm, respectively; *P* < 0.05). Moreover, by day 10, PEG hydrogel follicles had a significant increase in diameter compared to day 0 (day 0: 53.3 ± 0.5 vs day 10: 57.9 ± 1.0 µm; *P* < 0.05), whereas control follicles showed no difference (day 0: 52.9 ± 0.8 vs day 10: 55.0 ± 2.3 µm; *P* = 0.72). Follicles starting at 61–70 µm showed a decrease in diameter by day 10 of culture in control conditions (day 0: 64.7 ± 0.6 vs day 10: 56.7 ± 2.4 µm; *P* < 0.05), whereas follicles cultured in PEG maintained a similar diameter over time (day 0: 65.9 ± 0.6 vs day 10: 65.0 ± 1.8 µm; *P* = 0.68; Fig. 2C). Therefore, day 5 and 10 follicles were significantly smaller (*P* < 0.05) in control follicles when compared to similar timepoints of PEG follicles. Figure 2D shows a representative image of a freshly isolated follicle. Representative images of follicles grown in a PEG hydrogel over the 10-day period are depicted in Fig. 2E [25]. Due to difficulty in encapsulating small follicles, wells/hydrogels contained 1–4 follicles; as previous research has shown an advantage of groups culture for follicle growth [26], we did a separate analysis to determine whether there was an effect of single versus group culture in diameter change when the follicles were cultured in PEG hydrogels. We found that early primary follicles (41–60 µm) benefitted the most from group culture, whereas follicles transitioning between primary and early secondary stages performed similarly when encapsulated alone or in groups (Sup. Figure 1).

**Fig. 3:**
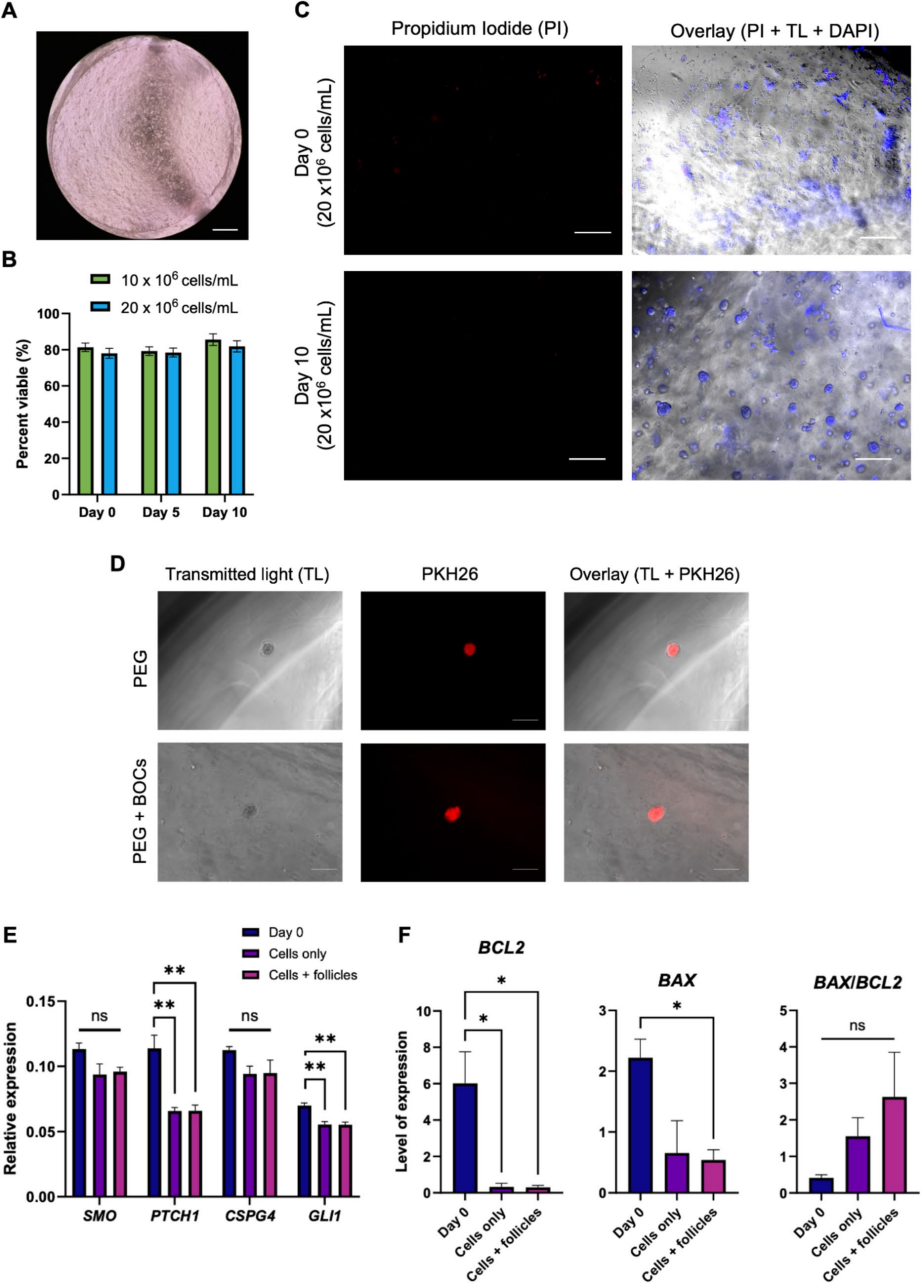
Co-culture of bovine preantral follicles with bovine ovarian cells (BOCs) in PEG hydrogels. **A** Representative image of BOCs in PEG hydrogel (scale bar = 500 µm). **B** Viability of BOCs encapsulated in PEG hydrogels during in vitro culture. **C** Propidium iodine (PI) staining in BOCs encapsulated in PEG hydrogels at day 0 and day 10 of culture. Counterstained with DAPI (TL, transluminescent; scale bar = 100 µm). **D** Unviable preantral follicle encapsulated in PEG hydrogel with or without BOCs using PKH26 to label follicles at day 10 of culture (scale bar = 100 µm). **E** Gene expression of pre-theca cell markers in BOCs (relative expression = 1/ΔCT). **F** Anti- and pro-apoptotic gene marker expression in BOCs (level of expression = absolute quantification from standard curve). Data are presented as mean ± SEM

Next, we evaluated cell viability of BOCs when encapsulated in PEG hydrogels and cultured for 10 days (Fig. 3A–C). When encapsulated at 1 or 2 × 10^6^ cells/mL, BOCs showed low cell viability (data not shown). Although higher concentrations of 10 or 20 × 10^6^ cells/mL showed high viability (70–90%) and cell aggregation (Fig. 3B and C), the high cell concentration made it difficult to visualize the follicles within the gel. Therefore, we evaluated preantral follicle growth in PEG hydrogels with and without the addition of 5 × 10^6^ BOCs/mL. We found that neither control (no BOCs encapsulated) nor hydrogels with BOCs supported follicle growth over the 10-day culture period (Fig. 3D). Moreover, gene expression analysis of pre-theca cell markers in the BOCs after 10 days of culture revealed a decrease in expression of *PTCH1* (*P* < 0.01) and *GLI1* (*P* < 0.01) when compared to day 0 fresh cells in both BOCs cultured alone and co-encapsulated with follicles (Fig. 3E). We found a decrease in both *BAX* and *BCL2* in BOCs that were cultured with follicles and a decrease in *BCL2* in BOCs cultured in PEG hydrogels alone (*P* < 0.05; Fig. 3F). However, there was no difference in *BAX*/*BCL2* ratio, thus indicating no difference in the pro- versus anti-apoptotic balance between cultured and fresh BOCs.

**Fig. 4:**
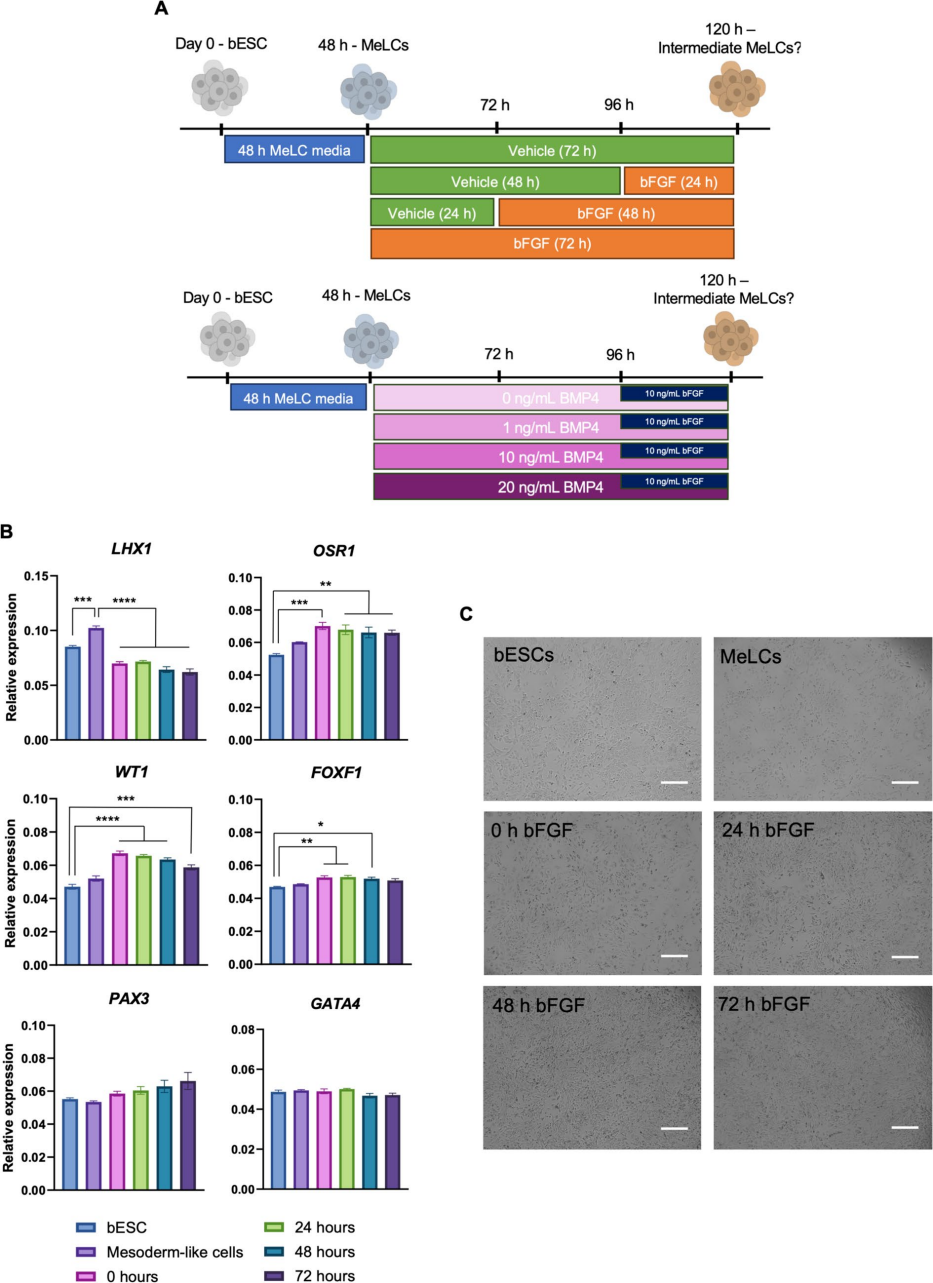
In vitro differentiation of bovine embryonic stem cells (bESCs) towards progenitors of somatic bipotential gonad-like cells. **A** Schematic diagram of experimental design for bFGF and BMP4 experiments. **B** Gene expression of intermediate mesoderm, bipotential gonad, paraxial and lateral plate mesoderm markers before and after bESC differentiation using bFGF at various days of exposure. **C** Representative images of cells from each treatment group. Data are presented as mean ± SEM. Significant differences are noted by *P < 0.05, **P < 0.01, ***P < 0.001, ****P < 0.0001. Figure made with Biorender

Given that native BOCs did not show maintenance of pre-theca cell identity or contribute to follicle growth, we then explored the differentiation of bESCs into progenitor cells of the bipotential gonad by testing the effect of WNT, bFGF, and BMP4 signaling (Fig. 4A). Developing a protocol to differentiate ESC to somatic gonadal cells would provide a consistent cell source for hydrogel complementation and avoid the variability of unknown ovarian cells, potentially contributing to future success of bovine preantral follicle culture. We first tested the hypothesis that MeLCs exposed to a pulse of bFGF during intermediate mesoderm/bipotential gonad differentiation would upregulate gene expression of key markers at the end of induction (Fig. 4B and C). Compared to bESCs and 0, 24, 48, and 72 h of 10 ng/mL bFGF exposure, MeLCs had higher expression of the early lateral plate/intermediate mesoderm marker *LHX1* (Fig. 4B; *P* < 0.001). We also found the intermediate mesoderm marker *OSR1* to be upregulated in all bFGF regimens compared to bESCs (*P* < 0.01). When analyzing expression of bipotential gonad markers, there was no difference in *GATA4* (*P* = 0.15) across experimental groups. We also found higher expression of *WT1* after 0, 24, 48, and 72 h of bFGF (i.e., after 4 additional days of culture) compared to bESCs and MeLCs (*P* < 0.001). There was no difference in expression of *WT1* between bESCs and MeLCs (*P* = 0.15). There was also no difference in expression of the paraxial mesoderm marker *PAX3* across all experimental groups (*P* = 0.06), indicating induction conditions did not show strong propensity to drive cells to the paraxial mesoderm fate. However, the lateral plate mesoderm marker *FOXF1* was upregulated compared to bESCs after 0, 24, and 48 h of bFGF exposure (*P* < 0.05).

**Fig. 5:**
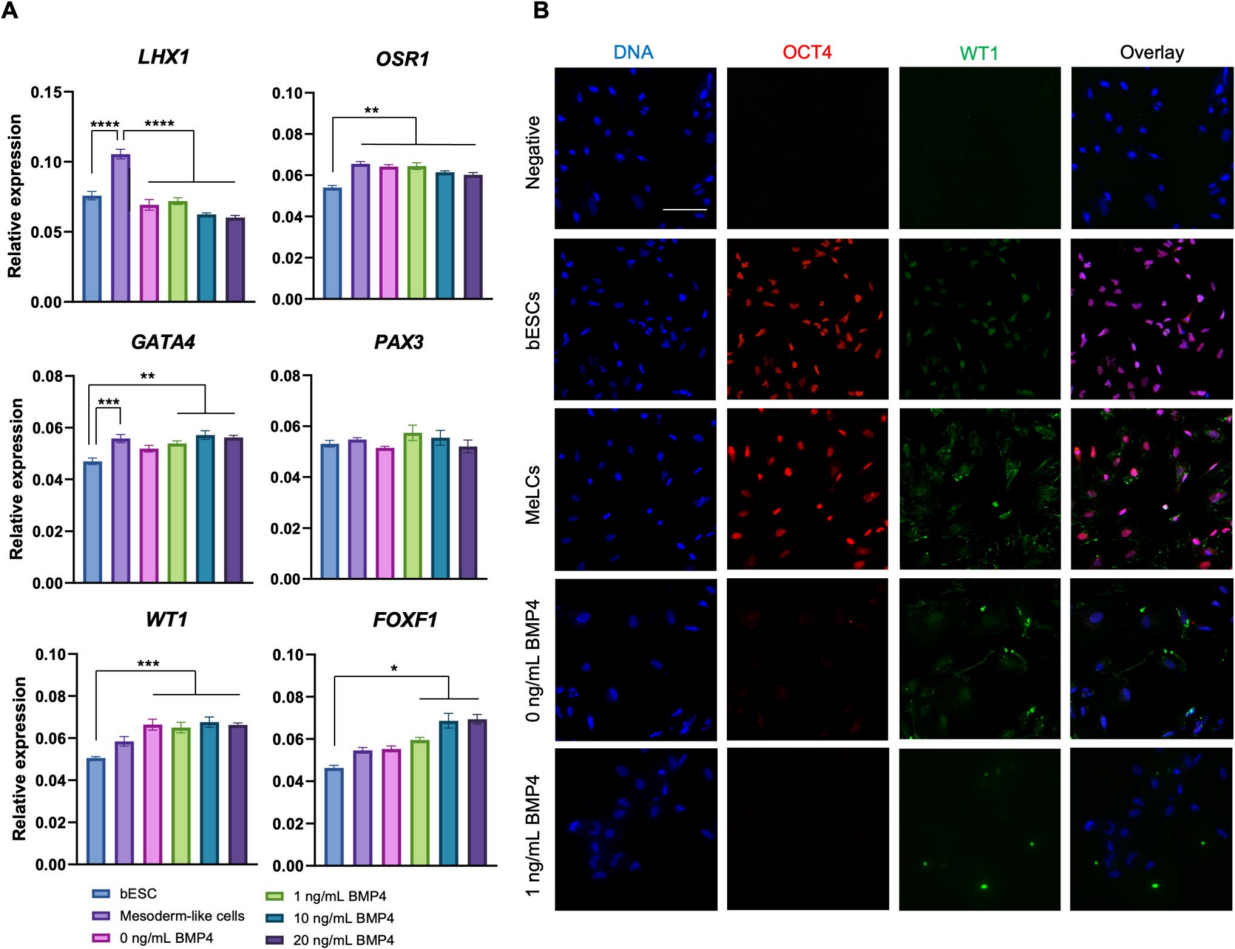
In vitro differentiation of bovine embryonic stem cells (bESCs) towards progenitors of somatic bipotential gonad-like cells testing BMP4 concentrations. **A** Gene expression of intermediate mesoderm, bipotential gonad, paraxial and lateral plate mesoderm markers before and after bESC differentiation. **B** Representative images of cells from each treatment group. Data are presented as mean ± SEM. Significant differences are noted by *P < 0.05, **P < 0.01, ***P < 0.001, ****P < 0.0001

Because all bFGF regimens tested caused elevation in *WT1* and *OSR1* expression, we chose to use a 24-h pulse of bFGF in the next experiments when testing the synergistic effects of including various concentrations of BMP4 over the same culture period time in two bESC lines (Fig. 5A and B). Similar to the bFGF experiment, we found the highest level of *LHX1* expression in MeLCs (Fig. 5A; *P* < 0.0001). However, 10 and 20 ng/mL of BMP4 led to lower expression when compared to bESCs (*P* < 0.05). Also confirming our previous results, *OSR1* was elevated in all experimental groups, including MeLCs, when compared to bESCs (*P* < 0.01). Unlike in the bFGF experiment where there was no difference amongst exposure times to bFGF, here we found an increase in *GATA4* in MeLCs and when 1, 10, or 20 ng/mL of BMP4 was present (*P* < 0.05). *WT1* was upregulated in 0, 1, 10, or 20 ng/mL BMP4, but not in MeLCs (*P* < 0.05). Again, we found no difference in *PAX3* expression among all concentrations of BMP4 when compared to bESCs. As expected, increasing the concentration of BMP4 led to a significant increase in *FOXF1* (*P* < 0.05), indicating a likely shift towards the lateral plate mesoderm lineage with rising levels of BMP4, mimicking in vivo development.

We further assessed WT1 and OCT4 expression in bESCs, MeLCs, and cells cultured with 0 and 1 ng/mL BMP4 as these concentrations led to elevated *WT1* without upregulating *FOXF1* (Fig. 5B)*.* We found a positive nuclear staining for OCT4 in bESCs and MeLCs and no signal in cells exposed to 0 or 1 ng/mL BMP4, indicating likely loss of pluripotency after mesoderm induction. We found nuclear localization of WT1 in bESCs and nuclear and cytoplasmic localization in MeLCs (48-h culture) and 0 ng/mL BMP4 (120-h culture). Interestingly, we did not observe WT1 expression at the protein level when cells were exposed to 1 ng/mL BMP4.

## Discussion

To our knowledge, this study is the first to utilize a novel bioengineered PEG hydrogel system tailored for in vitro culture of bovine preantral follicles and assess whether bESCs could be differentiated towards progenitor cells of the bipotential gonad using stepwise in vitro culture protocols. The ultimate goal of this work is to develop novel assisted reproductive technologies that enable the use of small preantral follicles in conjunction with precursor cells capable of becoming renewable theca-like cells for oocyte production. Since maintaining structural integrity of the follicle is a main concern with 2D culture, hydrogels represent a viable option as they provide framework to maintain the 3D configuration of follicles while permitting growth. Indeed, the theory to build a culture system complete with extracellular matrix components like the ovary has been suggested when early studies first explored bovine preantral in vitro development [27]. Further adaptations to include sophisticated ECM components in the hydrogel design have permitted mouse follicle growth in vitro starting as early as the primordial stage [28]. Our finding that ECM-degrading enzymes such as MMPs, PLAT, and PLAU are expressed by bovine preantral follicles agrees with previous investigations in follicles and spent medium [29, 30]. However, the fact that these transcripts were not uniformly expressed in all pools examined could indicate an intrinsic variability in the ability of the follicles to remodel the ECM and the PEG hydrogel. This could explain at least in part the variability observed in growth rate in the present study. Although we found that PEG hydrogels outperformed 2D control over 10 days in primary follicles, there was a notable difference in the percentage of follicles that grew between replicates (each represented by one ovary), further underlining the biological variability between samples and possibly an inherent propensity of some follicles for growth versus atresia. A limitation of this study using slaughterhouse-derived ovaries is the inability to track factors known to affect follicle viability such as animal age and nutritional and health status. Of note, early secondary follicles showed a stagnant rate of growth compared to primary follicles cultured in PEG hydrogels; this stagnation suggests that either (1) the 10 day-culture conditions remain substandard for bovine early secondary follicles or (2) follicles canonically slow their growth at this stage of development which is a phenomenon seen in mice [31]. It is worth noting that in vivo, 10 days represent a very narrow window within the estimated 180 days of folliculogenesis in the cow, and therefore, relatively small changes in growth could be expected in normally developing follicles.

We tested the hypothesis of enhancing the bovine follicle culture system with cellular supplementation as others have shown that the addition of ovarian stromal cells, fibroblasts, and adipose-derived stem cells promotes survival and growth of preantral follicles in other species [13–16, 32]. When BOCs were encapsulated in PEG hydrogels and cultured, we observed the formation of cell aggregates which may be due to the absence of basement membrane binding proteins in the PEG composition, rendering the cells unable to adhere to the matrix and resulting in self-aggregation. Indeed, cell-extracellular matrix interactions are important for mediating complex cellular responses such as migration, proliferation, and differentiation [33]; thus, BOCs may have been incapable of integrating well throughout the hydrogel. Furthermore, the finding of loss of expression of genes known to be important for the pre-theca cell phenotype after culture shows that BOCs did not maintain their identity, which could have contributed to the lack of success of this 3D co-culture system. Others have reported that mouse ovarian stromal cells transition from a predominately theca-like identity to almost entirely macrophage identity after 12 days of in vitro culture with follicles, agreeing with our findings of theca cell identity loss [15]. Since we found similarly high signs of atresia between follicles co-encapsulated and follicles in PEG-only hydrogels, we pose the possibility that the prolonged protocol required to dissociate ovarian tissue, isolate and label follicles, and process for encapsulation may have contributed to decrease follicle viability beginning at day 0. In future experiments, pre-theca cells could be sorted (pending availability of reliable antibodies against specific theca cell surface markers) before incorporation into the PEG hydrogel. Furthermore, culture conditions that specifically support theca cell phenotype would likely need to be investigated.

Since the addition of BOCs did not support follicle growth and the cells lost pre-theca cell identity in our culture system, we investigated whether bESCs could be differentiated towards a cell type mimicking pre-theca cells for future addition to the follicle culture system. This approach would circumvent the need to continuously isolate heterogenous ovarian cells since bESCs would be a renewable and consistent cell source to generate pre-theca-like cells for preantral follicle co-culture. We reasoned that this co-culture system could synergistically promote development of pre-theca-like cells and follicles via paracrine signaling as seen in mouse neonatal ovaries where Hedgehog signaling from preantral follicles drives the commitment of progenitor cells into the theca cell fate [34]. To achieve this goal, bESCs must undergo stepwise differentiation following the path of in vivo development to generate precursor gonadal cells. As a first step to achieve somatic gonadal cell differentiation, we used bESCs [24] to recapitulate mesoderm formation and early differentiation of the bipotential gonad with defined media conditions. We show that supplementation of the GSK3β inhibitor CHIR 99021 to enable WNT signaling can drive bESCs into intermediate mesoderm-like cells that show early signs of coelomic epithelial-like phenotype as indicated by gene and protein expression. During embryonic development, the sexually dimorphic gonad (ovary or testis) must develop beginning from the mesoderm germ layer and then further differentiate into the intermediate mesoderm and then coelomic epithelium which ingresses and gives rise to the sexually amorphic “bipotential gonad” [35, 36]. More specifically, upon Nodal, WNT, and FGF signaling to induce mesoderm differentiation [37], a subset of cells in the caudal aspect of the early gastrulated embryo are specified to become precursors of the bipotential gonad. Signaling by WNT, Nodal, and FGF during anterior–posterior (A-P) body axis extension, which occurs at peri-gastrulation and is regulated by TBXT and other morphogens in the mesoderm, drives cell fate decisions that result in caudal body axis formation [29, 30]. Concomitantly, mediolateral segmentation occurs when a low, medium, and high gradient of BMP signaling drives mesoderm cells into the paraxial, intermediate, and lateral plate mesoderm domains, respectively [38, 39]. Using this knowledge of developmental biology, we chose to modulate WNT, Nodal, FGF, and BMP signaling to mimic the molecular events that drive cell differentiation for pre-gonad development during early embryogenesis as shown in several mammalian species [36]. However, through these experiments, we found that under WNT activation, bFGF and BMP4 were not essential to induce the intermediate mesoderm or early coelomic epithelium in bESC. We observed peak expression of the intermediate mesoderm marker *LHX1* after 48 h of mesoderm induction under WNT and activin A, the latter a known inducer of *Lim1* (i.e. *Lhx1*) that has an important role in IM specification/kidney development [40]. We also found *OSR1* upregulated in all conditions after MeLC induction. *OSR1* is an early marker of IM and others have shown its necessity for the urogenital phenotype [41–43] and generating somatic cells of the gonad from PSCs [44]. Thus, we propose these cells may be reaching the IM state with possible capability of becoming progenitor bipotential gonad-like cell. Nevertheless, *OSR1* is not exclusive of the IM or gonads; kidney progenitor and lateral plate mesoderm cells express *OSR1* [43, 45] and this marker is used to demarcate kidney progenitor-like cells during in vitro differentiation of PSCs [46–48]. Therefore, our intermediate mesoderm/early coelomic epithelium-like cells should be further evaluated for markers of early nephrogenesis.

We additionally found a rise in *WT1*, which is also an early, and more restrictive, marker of gonadal progenitor cells [49], in induced cells across all bFGF and BMP4 regimens compared to bESCs. This indicates that continuous WNT activation for 3 additional days after initial mesoderm induction can induce *WT1* expression. WT1 is found along the A-P axis of mice as early as E9.0, with overlapping expression of GATA4 and NR5A1 in the coelomic epithelium by E10.0 and onward [49]. Moreover, WT1 activates the NR5A1 promoter [50], thus driving early gonad development. Interestingly, *WT1* upregulation was accompanied by *GATA4* upregulation when 10 or 20 ng/mL of BMP4 was included in culture which aligns with other studies [51]. However, we found variable expression of the protein WT1 in cells cultured beyond MeLC induction and after 3 additional days under 0 or 1 ng/mL of BMP4. Although typically found in the nucleus, WT1 isoforms with capability of shuttling between the nucleus and cytoplasm, therefore we postulate various WT1 isoforms with nuclear and/or cytoplasmic activity may be expressed in our cells [52]. We additionally highlight that there was no difference in *PAX3* in all experiments, demonstrating that our culture conditions did not favor differentiation of the paraxial mesoderm lineage. Interestingly, increasing BMP4 concentrations led to increased expression of *FOXF1* (lateral plate mesoderm marker), which aligns with in vivo studies showing a higher gradient of BMPs specifies the lateral plate of developing embryos [39].

Our findings provide seminal context to previous work looking at differentiation of ESCs from humans and mouse to gonad-like cells by using combinations of BMP4, FGF, CHIR, and activin A [53–55]. Moreover, this report is the first of its kind utilizing bovine ESC. Alternative approaches to inducing gonad-like cell formation include overexpression of key transcription factors such as *SF1*, *WT1*, *RUNX1*, and *GATA4* [56, 57]. The efficiency of induction can be variable when using small molecules or genetic engineering for differentiating stem cells; therefore, heterogeneity in cell identity would be expected in the present study. In the future, cell-sorting could aid in purifying cell populations of interest and used for downstream in vitro culture to achieve bipotential and even ovary or testis-like cells. Yet, in vivo early somatic progenitor cells of the bipotential gonad are not specified homogeneously; therefore, some heterogeneity could be warranted, especially when forming organoids as self-organizing structures. Finally, future studies should examine early bovine embryonic development in vivo as findings from these studies could shed light on the species-specific molecular mechanisms that drive gonad formation and inform future in vitro culture conditions.

In conclusion, our findings expand the current knowledge of in vitro preantral folliculogenesis using a non-rodent model. It also sheds light on methodologies for differentiating bESCs into precursor-like cells of the ovary which could be used in the future as a theca cell source for in vitro follicle culture. The convergence of these two technologies will be pivotal to broadening the knowledge base of follicle development and early gonadogenesis, expanding the potential for novel assisted reproductive technologies.

## Supplementary Information

Below is the link to the electronic supplementary material.

Supplementary file1 (DOCX 80 KB)

## Data Availability

The authors will provide raw data upon reasonable request.
