# Supplementary material for: Three-dimensional culture in a bioengineered matrix and somatic cell complementation to improve growth and survival of bovine preantral follicles
Source: J Assist Reprod Genet. 2025 May 20;42(5):1509–23. doi: 10.1007/s10815-025-03497-3 (PMC12167402; doi:10.1007/s10815-025-03497-3)
Supplement: Supplementary file 1 — Supplementary file1 (DOCX 80 KB) [file 10815_2025_3497_MOESM1_ESM.docx]

**Supplementary information**

**Supplementary Fig 1** Bovine preantral follicle in vitro growth in PEG hydrogels during single-follicle or group-follicle (n = 2-4 follicles) culture at different starting diameters. Data are presented as mean ± SEM.
